# Improved detectability of small-bowel lesions via capsule endoscopy with computed virtual chromoendoscopy: A pilot study

**DOI:** 10.3109/00365521.2011.584899

**Published:** 2011-05-30

**Authors:** Hiroki Imagawa, Shiro Oka, Shinji Tanaka, Ikue Noda, Makoto Higashiyama, Youji Sanomura, Takayoshi Shishido, Shigeto Yoshida, Kazuaki Chayama

**Affiliations:** 1 Department of Medicine and Molecular Science, Graduate School of Biomedical Science, Hiroshima University, Hiroshima, Japan; 2 Department of Endoscopy, Hiroshima University Hospital, Hiroshima, Japan

**Keywords:** Capsule endoscopy, flexible spectral imaging color enhancement

## Abstract

***Objective.*** Real-time video capsule endoscopy (CE) with flexible spectral imaging color enhancement (FICE) improves visibility of small-bowel lesions. This article aims to clarify whether CE-FICE also improves detectability of small-bowel lesions. ***Patients and methods.*** A total of 55 patients who underwent CE at Hiroshima University Hospital during the period November 2009 through March 2010 were enrolled in the study. Five patients were excluded from the study because residues and transit delays prevented sufficient evaluation. Thus, 50 patients participated. Two experienced endoscopists (each having interpreted more than 50 capsule videos) analyzed the images. One interpreted conventional capsule videos; the other, blinded to interpretation of the conventional images, interpreted CE-FICE images obtained at settings 1-3 (setting 1: red 595 nm, green 540 nm, blue 535 nm; setting 2: red 420 nm, green 520 nm, blue 530 nm; setting 3: red 595 nm, green 570 nm, blue 415 nm). Lesions were classified as angioectasia, erosion, ulceration, or tumor. Detectability was compared between the two modalities. Time taken to interpret the capsule videos was also determined. ***Results.*** Seventeen angioectasias were identified by conventional CE; 48 were detected by CE-FICE at setting 1, 45 at setting 2, and 24 at setting 3, with significant differences at settings 1 and 2 (*p* = 0.0003, *p* < 0.0001, respectively). Detection of erosion, ulceration, and tumor did not differ statistically between conventional CE and CE-FICE, nor did interpretation time (conventional CE 36 ± 6.9 min; CE-FICE setting 1, 36 ± 6.4 min; setting 2, 38 ± 5.8 min; setting 3, 35 ± 6.7 min). ***Conclusions.*** CE-FICE is superior in the lesion detection in comparison with conventional CE and improves detection of angioectasia.

## Introduction

The clinical usefulness of capsule endoscopy (CE) for diagnosing small-bowel diseases has been reported by various groups over the past 10 years [1-5]. Recently, we reported the usefulness of capsule endoscopy with flexible spectral imaging color enhancement (CE-FICE) for visualizing small-bowel lesions such as angioectasia, erosion/ulceration, and various tumors [6]. Our paper provided a statistical comparison of visualization by conventional CE versus CE-FICE as well as the rationale for use of CE-FICE over narrow band imaging and a summary of the basic FICE technology.

The promising results of this retrospective study led us to hypothesize that FICE would augment CE of the small bowel in such a way that would improve both detection and diagnosis of small-bowel lesions. We thus conducted a prospective study comparing detectability of small-bowel lesions on FICE-derived CE images with that on conventional CE images. Specifically, we asked whether the diagnostic capability of CE-FICE is superior to that of conventional CE for various small-bowel lesions.

## Patients and methods

### Patients

A total of 55 patients who underwent CE at Hiroshima University Hospital during the period November 2009 through March 2010 were enrolled in the study. CE was performed for the following reasons: obscure gastrointestinal bleeding (*n* = 34), examination of the extent of tumor spread (*n* = 8), investigation into the source of abdominal pain (*n* = 4), investigation into the source of chronic diarrhea (*n* = 4), close examination of inflammatory bowel disease (*n* = 3), suspected tumor (as indicated by computed tomography) (*n* = 1), and close examination for hypoproteinemia (*n* = 1). All 55 patients provided written informed consent for participation in the study.

### CE procedure

The CE capsule (PillCam SB2; Given Imaging Ltd, Yoqneam, Israel) was swallowed with a solution of dimethicone after an overnight fast, without any other preparation. Patients were allowed to drink clear liquids at 2 h and to eat a light meal at 4 h after swallowing the capsule. Images were analyzed with Rapid Reader 6 software on a RAPID 6.0 workstation (both from Given Imaging).

### FICE technique

As described previously [1], small-bowel video CE uses both a video capsule that contains an optical device to capture the images and a high-frequency transmitter that transmits high-quality images from the gastrointestinal tract to a portable data recorder, which is attached to a belt worn by the patient. The study is downloaded to the workstation and then reviewed using the RAPID software.

**Figure 1:**
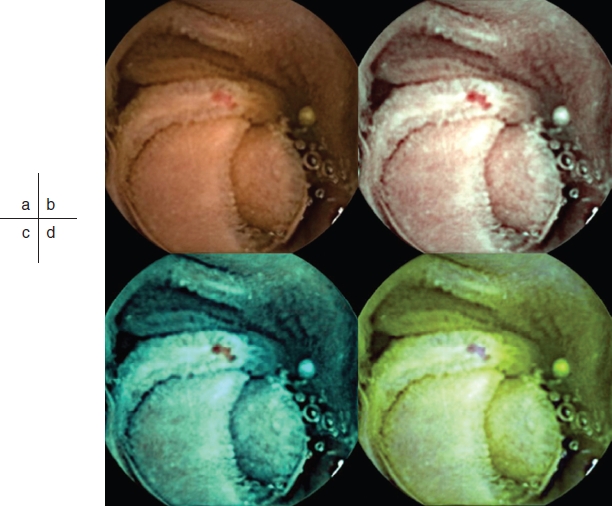
Example of an angioectasia. Although some reddening can be seen on the conventional image, the lesion is more clearly visualized by CE-FICE. a: Conventional CE image, b–d: CE-FICE images derived from the three different wavelength settings (b = setting 1; c = setting 2; d = setting 3). CE = conventional endoscopy; CE-FICE = CE with flexible spectral imaging color enhancement.

FICE can be used to enhance visibility of CE images. FICE is a spectral estimation technology based on arithmetic processing of ordinary images; application of FICE to CE does not require any re-engineering of the capsule device. The only requirement is integration of the FICE software into the computer workstation. The principle of FICE estimation technology is described elsewhere [7]. The wavelength spectrum used for creation of optical images is influenced by several factors: the spectrum of the light source, the optical device, and the spectral sensitivity of the sensing element. However, these factors differ between flexible endoscopy and CE, and therefore different FICE estimation algorithms with different estimation coefficients are required to optimize imaging. The spectral specifications (wavelengths) of the FICE settings that are useful in CE are as follows: setting 1: red 595 nm, green 540 nm, blue 535 nm; setting 2: red 420 nm, green 520 nm, blue 530 nm; setting 3: red 595 nm, green 570 nm, blue 415 nm [8] (Figure 1). With integration of the FICE digital processing system into the RAPID 6.0 workstation, it is possible to switch back and forth at any given time between the conventional CE image and the FICE image by a simple click on an icon at the RAPID software screen. The three different settings make it possible to select the most suitable wavelengths required for evaluation of the capsule video.

Small-bowel videos that were recorded by regular CE devices (PillCam SB2, Given Imaging) were evaluated on RAPID 6.0.

### Evaluation of CE-FICE images

The CE images were read by two experienced endoscopists who have interpreted more than 50 CE studies, and the same ability of reading and reading times. One endoscopist read the images obtained by conventional CE, and the other, blinded to the results of the conventional readings, read the images obtained by CE-FICE at settings 1, 2, and 3. The FICE reader read each FICE setting on another day and blinded to the results. Individual lesions were classified by both endoscopists as angioectasia, erosion, ulceration, or tumor. The numbers of lesions detected and reading times were compared between conventional CE and CE-FICE at settings 1, 2, and 3.

### Statistical analysis

The numbers of lesions detected by conventional CE and by CE-FICE at each of the three settings were determined, and differences were analyzed by Wilcoxon test; *p* < 0.05 was considered statistically significant. All analyses were performed with JMP software.

## Results

Five patients were excluded from the study because residues and transit delays prevented sufficient evaluation. Thus, the final study group comprised 50 patients (25 men, 25 women; average age, 59.6 years). Visualization of the entire small bowel was achieved in 74.0% (37/50) of cases, with gastric transit time being 22.9 ± 80.9 min and small bowel transit time being 307.4 ± 86.8 min in patients in whom the capsule passed through the entire small bowel.

**Figure 2:**
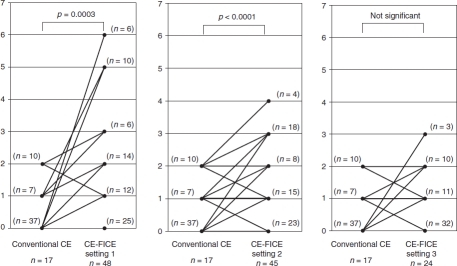
Increase in the number of angioectasias detected per FICE setting (vs. conventional CE). Abbreviations: CE = conventional endoscopy; FICE = flexible spectral imaging color enhancement.

Results of both conventional CE and CE-FICE are shown per lesion type in Table I. There was no statistical difference between conventional CE and CE-FICE in the detection of erosion, ulceration, or tumor. However, a total of 17 angioectasias were identified by conventional CE, but 48 were identified by CE-FICE at setting 1, 45 by CE-FICE at setting 2, and 24 by CE-FICE at setting 3. The difference in detectability of angioectasia was significant between conventional CE and CE-FICE at settings 1 and 2 but not setting 3 (Figure 2). A total of 20 erosive lesions were detected by conventional CE; 27 were identified by CE-FICE at setting 1, 33 at setting 2, and 31 at setting 3. The differences in detection between conventional CE and CE-FICE at the various settings were not significant. A total of 12 ulcerative lesions were detected by conventional CE-FICE; 13 were detected by CE-FICE at setting 1, 21 at setting 2, and 20 at setting 3. The differences in detection of ulcerative lesions between conventional CE and CE-FICE were not significant. A total of 40 lymphangiomas were detected by the conventional CE, and 45 were detected by CE-FICE at setting 1, 44 at setting 2, and 40 at setting 3. The differences were not significant. Finally, 15 tumors were detected by conventional CE; 16 tumors were detected by CE-FICE at setting 1, 13 at setting 2, and 13 at setting 3. The differences were not significant.

**Table I:** Number of lesions detected by conventional CE and CE-FICE.

		CE-FICE		
		
	Conventional	Setting	Setting	Setting
Lesion type	CE	1	2	3
Angioectasia	17	48*	45**	24
Erosion	20	27	33	31
Ulceration	12	13	21	20
Tumor				
Lymphangioma	40	45	44	40
Adenomatous polyp	1	1	1	1
Peutz-Jeghers polyp	7	7	7	7
GIST	5	6	4	4
Hemangioma	2	2	1	1

Abbreviations: CE = conventional endoscopy; CE-FICE = CE with flexible spectral imaging color enhancement; GIST = gastrointestinal stromal tumor.

* *p* = 0.0003 vs. conventional CE;

** *p* = 0.001 vs. conventional CE (Wilcoxon test).

Regarding particular types of tumor, 1 adenomatous polyp was detected by conventional CE, and the same adenomatous polyp was detected by CE-FICE at settings 1–3. The difference in the number of adenomatous polyps detected was not significant. Seven Peutz-Jeghers polyps were detected by conventional CE, but seven were detected by CE-FICE at setting 1, seven at setting 2, and seven lesions at setting 3; differences were not significant. Five gastrointestinal stromal tumors were detected by conventional CE; six were detected by CE-FICE at setting 1, four at setting 2, and four at setting 3. Again, differences were not significant. Detection of hemangiomas also did not differ significantly, with detection of two by conventional CE and of two by CE-FICE at setting 1, one at setting 2, and one at setting 3.

In terms of reading time, no significant difference was observed between conventional CE (36 ± 6.9 min) and CE-FICE at the various settings (setting 1, 36 ± 6.4 min; setting 2, 38 ± 5.8 min; setting 3, 35 ± 6.7 min) (Table II).

**Table II:** Capsule video reading times*

	FICE images		
	
Conventional CE images	Setting 1	Setting 2	Setting 3
36 ± 6.9	36 ± 6.4	38 ± 5.8	35 ± 6.7

Abbreviations: CE = conventional endoscopy; FICE = flexible spectral imaging color enhancement.

Differences did not differ statistically.

* Shown in minutes.

## Discussion

Detectability of colorectal tumors by means of FICE has been reported [10,11], but there are few reports regarding FICE for the source of bleeding in the small bowel. We used CE-FICE and found improved detectability of angioectasia at wavelength settings 1 and 2. Results with setting 3 were not promising. There was no improvement in detectability of erosion/ulceration or tumor. No lesions that were picked up by conventional CE were missed by CE-FICE. Thus, CE-FICE appears to be particularly useful for detecting small-bowel angioectasias. We reported previously that CE-FICE at settings 1 and 2 improves the visibility of angioectasia, erosion/ulceration, and tumor in the small intestine [6]. Specifically, we reported the following: At setting 1, improved visibility was achieved for 87% of angioectasias, 53.3% of erosions/ulcerations, and 25.3% of tumors. At setting 2, improved visibility was achieved for 87%, 25.5%, and 20.0%, respectively. We believe such improved visibility explains the improved detectability of angioectasias documented in the present study. Vascular lesions account for approximately 23-52% of cases of bleeding from the small bowel [11-13]; thus, improved detection of angioectasia would be clinically meaningful.

Application of FICE for visibility of various lesions in other organs has been recommended previously. We reported in 2009 that FICE, in comparison to white-light endoscopic evaluation, improved visibility of early gastric cancers by 46% [14]. Pohl et al. reported equal sensitivity between FICE and conventional chromoendoscopy with acetic acid for targeting biopsies in cases of high grade intraepithelial neoplasia/early cancer in patients with Barrett's esophagus [15]. Osawa et al. studied depressed-type early gastric cancer and reported that demarcation was easily identified by optimal band imaging without magnification in 96% of patients [16]. Yoshizawa et al. studied 81 elevated-type early gastric cancer lesions in 75 patients and reported that, compared to conventional endoscopy, FICE better depicted demarcation lines [17]. Togashi et al. reported that diagnosis of colorectal lesions on the basis of capillary patterns depicted by FICE was equal to or better than diagnosis based on pit-pattern analysis achieved with low-magnification chromoendoscopy [18].

Whether CE-FICE is advantageous needs to be evaluated. Reading time, for example, is an important clinical consideration. In our study, there was no statistical difference in reading time between conventional CE images and CE-FICE images. However, in actual clinical settings, it took twice as long to read CE-FICE images in addition to conventional CE images as it would have to read only CE-FICE images or only conventional CE images. In addition, there are three FICE settings to choose from, and it remains to be verified which of these settings is most appropriate. However, the fact that CE-FICE did not miss gross lesions such as tumors and ulcerative lesions is an advantage. With use of the QuickView component of RAPID 6.0 to preview the entire study, it is possible to improve the lesion detection rate. FICE may also be an option for beginners to reduce the likelihood of missing microlesions. An analysis of the surface structure of the lesion by FICE may also lead to qualitative diagnosis, as has been reported for colorectal tumors [19].

In conclusion, our data showed that CE-FICE is particularly useful for detecting angioectasias. We anticipate that the usefulness of FICE in cases of small-bowel bleeding will be further established by larger studies involving several institutions.
